# Advanced workstations and collaborative robots: exploiting eye-tracking and cardiac activity indices to unveil senior workers’ mental workload in assembly tasks

**DOI:** 10.3389/frobt.2023.1275572

**Published:** 2023-12-12

**Authors:** Patrik Pluchino, Gabriella F. A. Pernice, Federica Nenna, Michele Mingardi, Alice Bettelli, Davide Bacchin, Anna Spagnolli, Giulio Jacucci, Andrea Ragazzon, Leonardo Miglioranzi, Carlo Pettenon, Luciano Gamberini

**Affiliations:** ^1^ Department of General Psychology, University of Padova, Padova, Italy; ^2^ Human Inspired Technology (HIT) Research Centre, University of Padova, Padova, Italy; ^3^ Department of Computer Science, Helsinki Institute for Information Technology, University of Helsinki, Helsinki, Finland; ^4^ BNP Srl, Cittadella, Padova, Italy

**Keywords:** human factors, ergonomic workstations, collaborative robots, mental workload, psychophysiology

## Abstract

**Introduction:** As a result of Industry 5.0’s technological advancements, collaborative robots (cobots) have emerged as pivotal enablers for refining manufacturing processes while re-focusing on humans. However, the successful integration of these cutting-edge tools hinges on a better understanding of human factors when interacting with such new technologies, eventually fostering workers’ trust and acceptance and promoting low-fatigue work. This study thus delves into the intricate dynamics of human-cobot interactions by adopting a human-centric view.

**Methods:** With this intent, we targeted senior workers, who often contend with diminishing work capabilities, and we explored the nexus between various human factors and task outcomes during a joint assembly operation with a cobot on an ergonomic workstation. Exploiting a dual-task manipulation to increase the task demand, we measured performance, subjective perceptions, eye-tracking indices and cardiac activity during the task. Firstly, we provided an overview of the senior workers’ perceptions regarding their shared work with the cobot, by measuring technology acceptance, perceived wellbeing, work experience, and the estimated social impact of this technology in the industrial sector. Secondly, we asked whether the considered human factors varied significantly under dual-tasking, thus responding to a higher mental load while working alongside the cobot. Finally, we explored the predictive power of the collected measurements over the number of errors committed at the work task and the participants’ perceived workload.

**Results:** The present findings demonstrated how senior workers exhibited strong acceptance and positive experiences with our advanced workstation and the cobot, even under higher mental strain. Besides, their task performance suffered increased errors and duration during dual-tasking, while the eye behavior partially reflected the increased mental demand. Some interesting outcomes were also gained about the predictive power of some of the collected indices over the number of errors committed at the assembly task, even though the same did not apply to predicting perceived workload levels.

**Discussion:** Overall, the paper discusses possible applications of these results in the 5.0 manufacturing sector, emphasizing the importance of adopting a holistic human-centered approach to understand the human-cobot complex better.

## 1 Introduction

The rapid evolution of technology has had a profound impact on the manufacturing industry. Specifically, collaborative robotics (or cobotics) has gained attention for increasing accuracy and efficiency in manufacturing activities (Liu et al., 2022; Lorenzini et al., 2023). In this framework, innovations such as the Internet of Things (IoT) and the Industrial Internet of Things (IIoT) have enabled data-sharing between tools, sensors, and actuators, optimizing working activities and predicting maintenance needs (Wollschlaeger et al., 2017; Khan and Javaid, 2022). Artificial Intelligence (AI) is also being leveraged to enhance processes and ensure quality control (Jan et al., 2022; Morandini et al., 2023), while Big Data analytics is being used to identify trends and support supply chain management (Bag et al., 2020; Koot et al., 2021). Despite these technologies have a clear relevance for the manufacturing sector, their introduction into the industrial routines needs to be carefully implemented to avoid a low level of workers’ acceptance (Lu et al., 2022) and trust (Charalambous et al., 2016) towards such working technologies, that would otherwise result in a reduced use. Eventually, operators need to understand that these recent and advanced tools have not been considered as a replacement but instead as a support in carrying out the daily working activities.

The shift in the conceptualization from Industry 4.0–5.0 has in fact brought to light the centrality of human beings, their individual characteristics and needs (e.g., ageing and consequent physical or cognitive decline). In this view, besides the strong interest in the digital transition, the introduction of cutting-edge hardware and software solutions and AI-driven technologies must be carefully considered, on the one hand, to support efficient and flexible industrial productivity, and on the other hand, to back individuals and society. To pursue the latter point, technologies must adapt to the needs and individual features of industrial workers (Lu et al., 2021; Lu et al., 2022), while adhering to the principles of social fairness and sustainability inherent in Industry 5.0 (Xu et al., 2021; Huang et al., 2022; Ivanov, 2023). This human-centric approach is also endorsed by the European Commission (Breque et al., 2021) and is essential for creating accessible, inclusive, and safe working environments that enhance physical and mental health, wellbeing, and the quality of working life.

In the manufacturing sector specifically, advanced ergonomic workstations and collaborative robots play a pivotal role in this shift toward a human-centric focus. These enabling technologies are designed to work alongside human operators, providing ergonomic features and promoting user-centered design (Panchetti et al., 2023). In cobotics, operators and cobots share time and workspace, directly interacting to perform tasks (Hopko et al., 2022). The introduction of these technologies typically increases acceptance, intention of usage, and actual usage among end users (Weiss and Huber, 2016; Meissner et al., 2020).

These advanced workstations offer various ergonomic features, such as adjustable height, smart lighting, pick-to-light systems, and torque reaction arms to improve the operator’ comfort, safety and acceptability. Furthermore, there are relevant differences between traditional robots and cobots. For instance, traditional industrial robots do not allow the human-robot direct physical interaction, and therefore do not need any safety features to ensure the physical integrity of the worker. Differently, cobots allow a shared workspace and close actions of humans and cobots. To ensure workers’ safety when closely interacting with these technologies, cobots are equipped with several sensors (e.g., proximity, smart cameras) and safety features (i.e., force and speed limiting and collision avoidance systems; Sherwani et al., 2020). This heightened level of safety measures enables cobots to interact securely with human workers in close proximity. They effectively bridge the divide between the physical limitations that traditional industrial robots entail. By assuming responsibility for physically demanding and repetitive tasks while concurrently minimizing the risks of errors, waste, injuries, and accidents, cobots reveal their substantial advantages for human workers, with particular significance for senior workers.

The human-cobot framework is thus characterized by a symbiotic relationship that combines human expertise, creativity, and the ability to handle unforeseen situations, in conjunction with the precision and unwavering performance of robots. According to Kopp et al. (2021), the effectiveness of a human-cobot dyad can be influenced by three elements: worker’s skills, cobot performance, and their mutual interaction. Remarkably, there is recent literature that highlights how, by bringing the focus on humans within the human-cobot interplay, the study and assessment of human factors become essential. For instance, Paliga and Pollak (2021) and Paliga (2022), proposed the concept of fluency in human-robot collaboration, which seeks to replicate the seamless interactions observed in human teams. Furthermore, physical ergonomics, trust, acceptance, user experience and usability of these working tools, and the level of operators’ mental workload are crucial aspects that must be measured in order to introduce cutting-edge workstations and collaborative robotics in the workplace effectively (for a review, see Faccio et al., 2023). All these factors, along with the aging factor that is central in our investigation, are detailed in the following paragraphs.

Concerning **physical ergonomics**, researchers such as Gualtieri et al. (2021) and Gaultieri et al. (2020a) have explored the assessment of anthropometric data, focusing on workbench heights and the positions of tools, including dispensers. To accomplish this, both virtual and real prototypes have been employed to determine the correct positioning of workers’ arms, shoulders, and backs, often using wearable devices. The objective was to mitigate the risk of musculoskeletal problems (Colim et al., 2021a). In certain scenarios, these ergonomic risks can be alleviated by delegating specific assembly phases to robotic counterparts, thereby reducing the strain on operators’ hands and wrists (Colim et al., 2021b). This approach not only lessens physical fatigue but also optimizes overall body posture (Lorenzini et al., 2019). Further research underscored the significance of task allocation and increased collaboration with cobots in comparison to entirely manual work processes (Liau and Ryu, 2020). Ultimately, reducing physical risks for workers can be achieved by entrusting manual handling of heavy components and repetitive tasks to collaborative robots (Gualtieri et al., 2020b; Cardoso et al., 2021).

Other fundamental factors to account for are **trust and acceptance** of cobots (Rossato et al., 2021a; Panchetti et al., 2023). In fact, these working tools can be seen as a threat or an opportunity. The former can lead, for example, to a reduction in work motivation related to the fear of employment loss, while the latter can be characterized, for instance, by a decrement of physical and mental strain (Meissner et al., 2020). Furthermore, research suggests that cobots must be related to a positive working experience and characterized by high levels of usability to influence the perceptions of workers favorably (Hopko et al., 2022; Faccio et al., 2023), for example, by permitting the workers to customize the cobot behavior (e.g., speed, type of interaction; Fraboni et al., 2022) or choosing the interaction modality (i.e., direct physical interaction or mediated by a control interface; Rossato et al., 2021a). Nonetheless, so far, there are more studies focusing on the acceptance of healthcare and assistive robots but yet not enough research in the industrial domain (Savela et al., 2018).

Concerning the **human mental/cognitive workload** (Van Acker et al., 2018), previous studies have quantified this factor by processing various psychophysiological indices that can affect human-cobot interactions or by collecting and analyzing self-reports. For instance, some researchers have considered indices related to eye behavior, such as fixation duration/number (Matthews et al., 2015; Wu et al., 2020) or blink rate/duration (Nenna et al., 2023). Others have analyzed cardiac activity, for example, heart rate or heart rate variability (Charles and Nixon, 2019; Lagomarsino et al., 2022; Lin and Lukodono, 2022), which can reflect fluctuations in the level of mental workload while performing working tasks. To explore the mental workload in experimental settings, the scientific literature has outlined how the manipulation of experimental tasks (e.g., dual task, time pressure, etc.) can induce elevated levels of mental load and negatively influence participants’ performance and subjective experiences (Galy and Mélan, 2015; Shaw et al., 2018; Vasquez et al., 2019). Similarly, the subjective perception of participants’ cognitive workload (i.e., NASA-TLX; Chacón et al., 2021; Rossato et al., 2021a) or the decrement in work performance are also typically used for measuring the human mental/cognitive load. For instance, longer time on task or higher error rate are indicative of increased mental demand (Rossato et al., 2021a; De Simone et al., 2022; Fraboni et al., 2022; Panchetti et al., 2023).

Finally, considering the extension of working life, the **age of operators** (i.e., >50–55 years) is a human factor that is recently gaining increasing importance. This element can significantly influence operators’ perception and interaction with cutting-edge working tools such as cobots. Several studies have investigated the senior workers-cobot interaction and overall experience (Bogataj et al., 2019; Rossato et al., 2021a). Bogataj et al. (2019) outlined the need to invest in workplace ergonomics and cobots to reduce the fatigue and mental stress of old operators, which can mitigate the decrement in their working abilities (e.g., speed, physical strength). A recent literature review (Calzavara et al., 2020) described several benefits related to the introduction of cobots considering the management of ageing workforce. Specifically, they mentioned how simplifying tasks, assigning to cobots the non-ergonomic activities, and enhancing the quality of work output (i.e., human-cobot co-monitoring) are the most beneficial aspects. Rossato et al. (2021a) showed that senior workers perceived the cobot as more supportive than a sample of adult workers. These last reported high levels of satisfaction, cobot’s perceived ease of use, and besides high pleasantness when they had the opportunity of interacting physically with it. Recent studies (Rossato et al., 2021a; Rossato et al., 2021b), reported various primary elements that can affect the aged operators’ acceptance of advanced workstations equipped with cobots, such as perceived utility, sense of safety, and the need for proper training to use these technologies. Indeed, operators’ ageing can make it difficult to ensure high knowledge and skills to deal with advanced technologies.

Taking all this, the main objectives of the present study are to: a) evaluate the subjective perceptions of senior workers in terms of technology acceptance (before and after both post-tests), perceived wellbeing and working experience with an advanced workstation and a cobot and the estimated social impact of this integrated working technology in the industrial sector; b) assess whether the human factors considered in the present research (i.e., task performance, subjective perceptions, eye tracking indices and cardiac activity) variate significantly under dual-tasking (i.e., under higher mental load); c) explore the predictive power of the collected measurements over the number of errors committed at the work task and over the perceived mental demand. For clarity, we have provided a table (i.e., Table 1) collecting all the acronyms used along the paper.

**TABLE 1:** Acronyms used along the paper.

Full term	Acronym
Collaborative robots	cobots
Internet of Things	IoT
Industrial Internet of Things	IIoT
Collaborative robotics	Cobotics
Artificial Intelligence	AI
Assistive Assembly System	AAS
Smart Manufacturing Manager	SMM
Hardware	HW
Software	SW
Heart Rate	HR

## 2 Materials and methods

The study was carried out with ethical committee approval by the Ethics Committee of the Human Inspired Technology Research Centre (HIT) (Protocol number: n.2019_58).

### 2.1 Participants

Fifteen workers (*M*_age_ = 55.21, *SD*_age_ = 3.65, F = 4) were recruited for the experiment. The inclusion criteria were that the age was equal to or higher than 50 years old, normal or corrected-to-normal vision, and no heart diseases, and that participants were active workers in the industrial domain. Participants received compensation for partaking in the trial (i.e., 25 euros). Eleven participants (*M*_age_ = 54.72, *SD*_age_ = 4.05, F = 4) were considered in the statistical analyses. Indeed, four participants were excluded for low accuracy of the eye-tracking and/or cardiac activity data. Participants were recruited by an agency, that was a sub-contractor of the Co-Adapt H2020 EU project, with experience in recruiting senior workers within the industrial/artisanal sectors.

### 2.2 Experimental design

A within-participants design was adopted for the experiment. All participants had to accomplish a single and a dual task (counter-balanced order). Following a dual task paradigm, we manipulated the task difficulty (i.e., independent variable) by adding a secondary task (i.e., mathematical) to the main one (i.e., assembling task).

### 2.3 Tasks

In the single task condition, participants had to accomplish an assembly task conceived in collaboration with BNP Srl company to have an ecological working activity carried out in a laboratory setting. Typically, an assembly task is a manufacturing or production process that involves the assembly of various components, parts or materials to create a finished products or subassembly. The same assembly task was performed four consecutive times.

**FIGURE 1:**
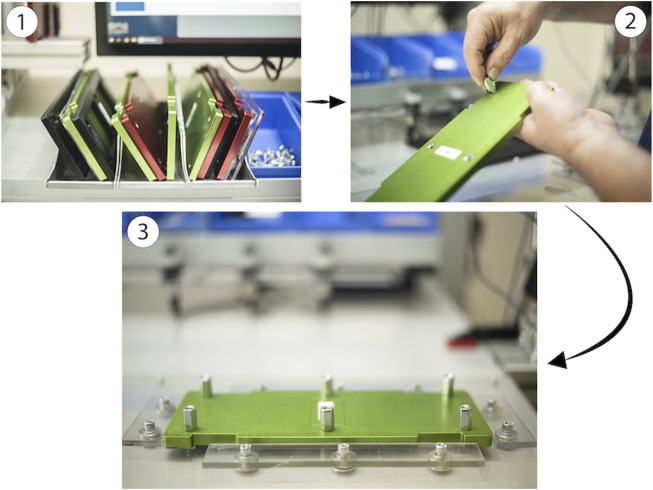
Participants had to choose the metallic plate (1), tighten the screws (2), and place the first plate on the AAS workbench (3).

In the first step, following the instructions presented on the monitor, participants had to choose a green plate (step 1; i.e., a green metallic plate, “*Retrieve a green plate*”) and manually tighten the screws (step 2; i.e., “*Pick up and screw in six pillars and six screws onto the green plate, taking them one at a time from the steel tray. Ensure with your hands that all six pillars are tightened*”). Next, they had to place the plate inside a specific area delimited by pieces of plexiglass tightened with screws on the workbench (step 3; i.e., “*Position the green plate between the stops on the right side of the table. Press the “next” button at the top right of the workstation screen*”; Figure 1).

**FIGURE 2:**
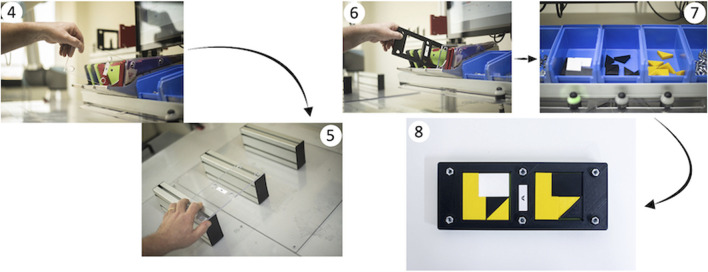
Participants chose a transparent plate (4), placed the plate in the cobot area (5), picked up the plastic plate (6) and pieces (7), and formed a puzzle (8).

Then participants had to choose a transparent plate (step 4; i.e., “*Take a transparent plate*”) and place it in the cobot’s area (step 5; “*Place it below the cobot on the supports*”) on two supports. Following, they had to pick up a black plastic plate (step 6; i.e., “*Retrieve a black plastic mold with the same letter as the green plate*”) and a set of colored plastic pieces (step 7) to form a puzzle (step 8; i.e., “*Complete the puzzle using the components in the blue boxes following the pattern in the illustration*” Figure 2), while the cobot pretended to glue the transparent plate.

**FIGURE 3:**
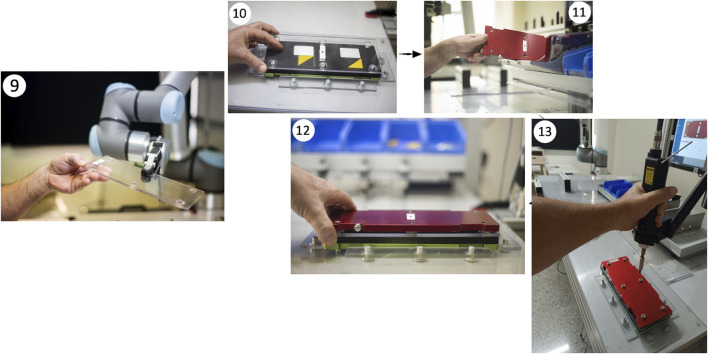
Cobot passed the plate to the participants (9), they placed the plate on the semi-assembled piece (10), took a red metallic plate (11), and tightened the screws (12–13).

When the cobot finished its task, it passed the transparent plate to the participants (step 9; i.e., “*Take the transparent cover that the robot brought to you. Press the “next” button at the top right of the workstation screen*”). They placed it on the semi-assembled block of components (step 10). Finally, senior workers picked a red metallic plate (step 11; i.e., “*Retrieve a red plate with the corresponding letter and position it above the semi-assembled block of components and press the “next” button at the top right of the workstation screen*”) and used an electric screwdriver to tighten the screws following a specific sequence detailed in the instructions presented on the Assistive Assembly System (AAS) monitor (step 12–13; i.e., “*Retrieve the screws one at a time from the steel tray and perform the screwing in the indicated order and use the screwdriver located on the arm to your right and press the finish button when you have completed the screwing task*”). The final assembled object is depicted in Figure 3 (step 13).

In the dual task condition, participants had to carry out a mathematical task aloud simultaneously. They had to subtract seven from 800 and again from the result until the main assembly task was completed (4 times). We asked participants to be accurate and fast as much as possible while performing both experimental tasks. A familiarization phase (see Procedure Section 2.5 for details) was considered for both types of tasks (i.e., assembly and mathematical).

### 2.4 Equipment and materials

**FIGURE 4:**
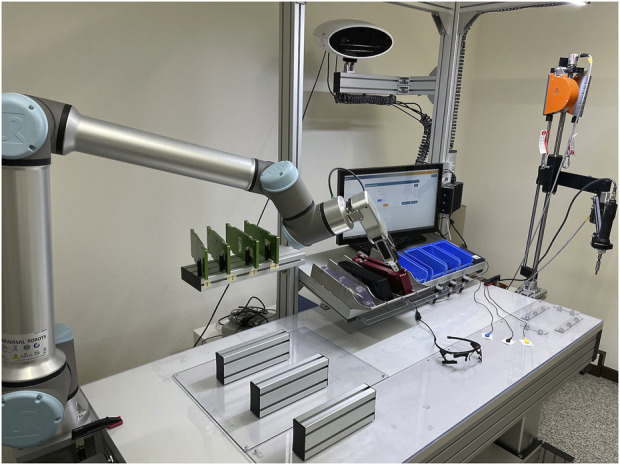
The Assembly Workstation with cobot, eye tracker, and surface electrodes on the workbench.

An advanced workstation equipped with a collaborative robot (Assistive Assembly System; AAS) was exploited in the experiment. The integrated working tool is graphically depicted in Figure 4. The collaborative robot is installed aside from the workbench.

The AAS comprises several hardware (HW) and software (SW) components as follows:

HW:

• collaborative robot (Universal Robot UR10e) with its teach pendant (control interface);
• adaptive workbench with adjustable height;
• smart lighting system;
• gesture detection and safety smart camera;
• LCD touch screen (on which the task instructions were displayed);
• force reaction system and a comfortable electric tightener;
• RGB Pick-to-Light smart system;
• Wearable eye-tracking glasses;
• Amplifier and non-invasive surface electrodes for monitoring cardiac activity.

SW:

• Smart Manufacturing Manager (SMM), offering real-time interactive multimedia instructions;
• Integration of the eye-tracking and physiological software API/SDK for synchronizing the data acquisition.

We utilized a collaborative robot (UNIVERSAL ROBOT; UR10e) which adheres to a stringent set of safety standards as outlined in ISO/TS 15066:2016, making it suitable for operation in close proximity to human workers. It boasts a considerable payload capacity of 10 kg and exhibits remarkable versatility in reaching diverse positions on the workbench. The robot system comprises a robotic arm, complemented by a user-friendly interface installed in a “teach pendant”, i.e., a tablet device. This interface empowers users to establish virtual boundaries around the cobot, serving as a proactive safety measure to prevent inadvertent collisions with other objects or surfaces. Besides, the UR10e is equipped with an automatic safety feature that halts its movement if any attempt is made to breach these pre-defined safety boundaries. In this particular setup, we employed a gripper as the end effector, facilitating the robot’s capability to grasp and release assembly components. The teach pendant permitted the programming of all the cobot movements in the collaborative task.

Besides, participants wore a pair of eye-tracking glasses (i.e., Pupil Labs; maximum sampling frequency of 120 Hz, accuracy of 0.5 visual angle degrees) during the whole experimental session. This tool allowed the collection of fixations and blinks data (i.e., duration and frequency) and pupil diameter. An MSI laptop (Intel Core i7-6700HQ, screen resolution 1920 × 1080) was connected using a USB cable to the eye-tracking glasses, permitted to perform the calibration phases and store the eye-tracking data.

A portable amplifier (ProComp5 Infiniti; ^©^ 2022 Thought Technology Ltd.) and its software (i.e., BioGraph Infiniti; ^©^ 2022 Thought Technology Ltd.) installed on a second MSI laptop (Intel Core i7-6700HQ, screen resolution 1920 × 1080) were utilized to gather physiological data related to the cardiac activity. The amplifier comprises five channels (i.e., it can record up to 2,048 Hz). For the present purpose surface electrodes were used, and the sampling rate was set at 256 Hz.

Two 4K cameras (Value HD Corporation^©^) were positioned in the laboratory to allow the video recordings (acquired with the software WMIX HD Edition) of all the experimental sessions.

Recording user interactions with technology in complex settings, such as workplaces, has been widely embraced (Heath and Luff, 2018; Blackler et al., 2018). Researchers can scrutinize the recorded behaviors pertinent to their investigations, employing lucid, observable criteria to ensure impartiality and mitigate bias (Bakeman and Quera, 2012; Guo et al., 2015).

The WMIX HD Edition software was employed to process and synchronize footage captured by each camera resulting in a unified video. Subsequently, these videos were imported into BORIS software, and a coding scheme was independently devised by two researchers based on the observed behavioral patterns. Discrepancies were addressed through discussion, thereby diminishing subjectivity. Subsequent analysis, executed with the concurred-upon coding framework, revealed that certain manual errors (e.g., errors in selecting the correct plates; screw tightening sequence) were infrequently committed by participants. For this reason, the errors were included in a single final category. The refined coding scheme is presented in Table 2.

**TABLE 2:** Coding scheme.

Coded event	Description
Time on task	The time spent in completing each task (i.e., single task, dual task)
Error in the assembly process	These errors occurred each time the participant failed in some operations of the assembly process
Error in the math task	These errors occurred each time the participant failed in the -7 task

The following self-reported tools were administered:

• Demographic questionnaire (PRE), this tool aimed at collecting background information (e.g., gender, age, experience with collaborative robots, etc.).
• NASA-TLX (POST), the NASA-Task Load Index (Hart and Staveland, 1988; Hart, 2006) was used for assessing the task load in the different experimental sessions. This tool comprises the following six sub-scales: mental, physical, and temporal demands, perceived performance, effort, and frustration. Each sub-scale presents a response based on a 20-step bipolar scale (i.e., range: 5–100). It is possible to evaluate each scale (Galy et al., 2018) independently or consider an overall score by merging the scores of the individual scales.
• TAM 3 (PRE-POST). We adapted the TAM3 questionnaire (Venkatesh and Bala, 2008) considering 16 items and the following constructs: Perceived Usefulness (PU; 4 items), Perceived Ease of Use (PEOU; 4 items), Perception of External Control (PEC; 3 items), Perceived Enjoyment (PE; 3 items), and Behavioral Intention (BI; 2 items). All items were measured on a 7-point Likert scale (i.e., from 1, strongly disagree, to 7, strongly agree).
• *Ad hoc* wellbeing and working experience questionnaire (PRE-POST). This instrument comprises a total of 14 items considering the following dimensions: work satisfaction (4 items), motivation (3 items), engagement (3 items), and overall working experience (4 items). A 5-point scale was used to respond (i.e., from 1, not at all, to 5, extremely).
• Social impact (PRE-POST). This dimension was assessed utilizing a single item (Gervasi et al., 2020). We asked, “which will be the introduction of our workstation in the industrial sector?”. The response options were: it will cause the dismissal of workers; it will positively affect the working activities but it will not cause the dismissal of workers; and it will not produce any effect on the working activities.

### 2.5 Procedure

The experimental sessions were carried out in a quiet and isolated laboratory. Upon participants’ arrival they were administered with the informed consent and an informative note. They had to fill out a battery of pre-test questionnaires (i.e., demographic, TAM 3, Social impact, and Wellbeing and working experience).

According to Rossato et al. (2021a), the height of the workbench, where participants were asked to accomplish the various tasks, was meticulously adjusted to conform with precise ergonomic standards, such as ensuring that the workbench’s height corresponds to the height of the bent elbow aligned parallel to the ground, minus 150 mm. Afterward, participants were asked if they found comfortable the workbench height to reach various locations shown by the experimenter, that were linked to the actual assembly activity and to use the tools of the workstation (e.g., electric tightener). Thus, in case of an affirmative response the first set of pre-recorded instructions was presented. This information was provided prior to each experimental condition. Nevertheless, researchers were available to clarify any doubt to participants.

A familiarization phase (10–15 min) allowed participants to learn how to perform the assembly task utilizing the cobot, the Smart Manufacturing Manager (SMM) control interface, and the electric tightener. Following this, the experimenter helped participants wearing eye-tracking glasses, and three non-invasive surface electrodes were placed on their chests. The eye tracker was calibrated following a standard procedure using external markers. Afterward, participants were still maintaining their gaze on a cross made of two tapes that were located on a wall at a specific distance from the chair (2.5 m). This phase was carried out to acquire the baseline of their gaze behavior and cardiac activity in a resting condition. The baseline permitted in the pre-processing phase to set the threshold for considering an eye closure as a blink and to avoid artifacts in the data (e.g., not a real blink but a moment in which the eye was ajar).

**FIGURE 5:**
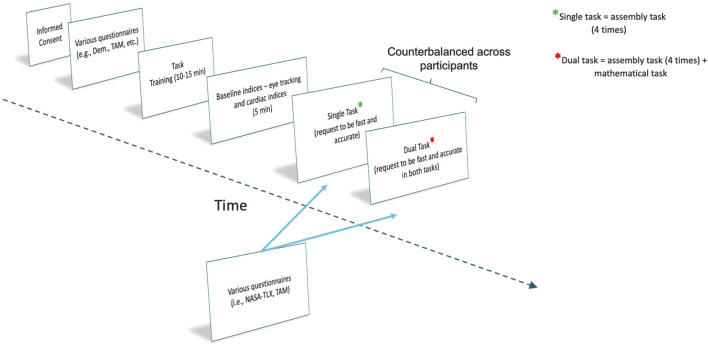
Experimental procedure.

After the baseline, participants began the experimental tasks. The first condition (e.g., single task) was equal for all participants. They had to perform an assembly activity utilizing the AAS equal to the task of the familiarization phase four consecutive times. In the second condition (e.g., dual task), participants had to simultaneously perform a secondary task: mathematical counting. Participants while performing the assembly task, had to simultaneously subtract seven from 800 and again from the obtained result up to when they accomplished 4 times the assembly task. At the end of each condition, a set of questionnaires was administered. Participants had to complete the NASA-TLX, TAM3, and the Social Impact questionnaire. Single and dual task were counterbalanced across participants (i.e., a sub-group of participants performed first the single task, and then the dual task the other sub-sample first accomplished the dual task and then the single task). Participants carried out the various assembly tasks without speed pressure or a pre-specified time interval. Additionally, to mitigate fatigue-related effects, scheduled intervals of rest were incorporated between the completion of the questionnaires and the beginning of the subsequent task, which were tailored to the needs of each participant. The overall experiment lasted around 45 min. A graphical depiction of the procedure is presented in Figure 5.

### 2.6 Measures

The following dependent variables were considered, related respectively to performance, subjective perceptions, eye behavior, and cardiac activity:

• Performance (i.e., nº of errors in the assembly and percentage of accuracy in the mathematical task, time on task in sec);
• Pre- and Post-test questionnaires scores (e.g., NASA-TLX, acceptance, wellbeing and working experience);
• Fixations duration (ms) and frequency (min);
• Blinks duration (ms) and frequency (min);
• Heart Rate (HR; bpm).

## 3 Results

For the sake of brevity, in the following sections, only the analyses that showed significant differences among the experimental conditions are reported. All analyses were conducted using the software RStudio (R Core Team, 2022).

In the case of data normally distributed, ANOVA analyses were performed. Differently, non-parametric (i.e., Wilcoxon tests) analyses were considered, and the Benjamini and Hochberg (1995) correction was applied to adjust *p*-values. Regarding the parameters enclosed in parentheses, we provide the following explanations for clarity: *t/V* = respectively the value of a t-test or Wilcoxon test; *d* = Cohen’s d effect size value; *r* = effect size value for Wilcoxon tests (Field et al., 2012; Page 665); and *R* ^2^ = r-squared of the model.

### 3.1 Performance

#### 3.1.1 Errors

**FIGURE 6:**
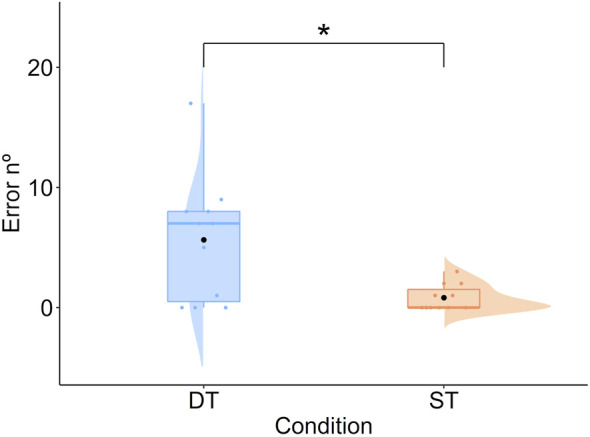
Mean number of errors in the assembly task as a function of condition. Note: DT, Dual Task; ST, Single Task.

A difference emerged (*t* = −2.87, df = 10, *p* < 0.05, *d* = 1.28). Participants committed a higher number of errors in the dual task (*M* = 5.64) compared to the single task (*M* = 0.81; Figure 6). On average, participants performed well on the secondary task (*M* = 77.23%), reflecting the mental workload imposed by the dual task condition.

#### 3.1.2 Time on task

**FIGURE 7:**
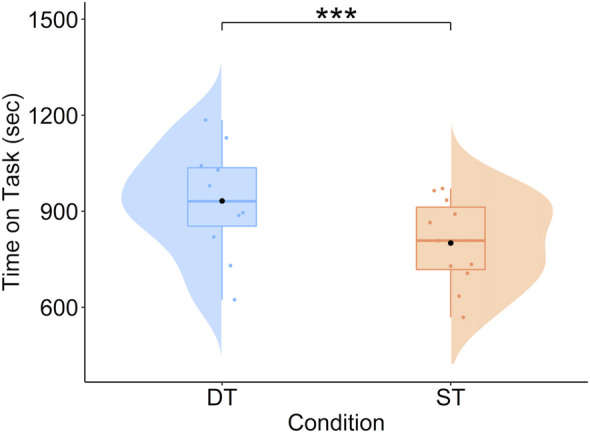
Mean Time on Task (seconds) in the assembly task as a function of condition. Note: DT, Dual Task; ST, Single Task.

A difference emerged (*V* = 0, *p* < 0.001, *r* = 0.99). Participants were faster in performing the single task (M = 806.82 s) compared to the dual task (*M* = 966.84 s; Figure 7).

### 3.2 Subjective perceptions

#### 3.2.1 NASA-TLX

Several Wilcoxon tests were carried out considering the NASA-TLX sub-scales (20-step bipolar scale, range: 5–100). Regarding the mental demand sub-scale, a difference was highlighted (*V* = 0, *p* < 0.05, *r* = 0.87). Participants reported a higher level of mental load in the dual task (*Mdn* = 100) compared to the single task (*Mdn* = 45). Besides, considering the effort sub-scale, a difference was shown (*V* = 0, *p* < 0.05, *r* = 0.79). Senior workers reported a higher effort in the dual task (*Mdn* = 95) compared to the single task (*Mdn* = 60).

#### 3.2.2 TAM

No differences emerged considering all the TAM dimensions (all *p*_*s*_ > 0.05). Nevertheless, Table 3 shows that all the mean and median (in parentheses) scores were above the scale median (i.e., 4; 7-point Likert Scale, strongly disagree-strongly agree).

**TABLE 3:** TAM questionnaire dimensions and scores.

TAM dimensions	Pre-test	Post ST	Post DT
	Mean (Median)	Mean (Median)	Mean (Median)
Perceived Enjoyment	4.27 (4)	5.45 (6)	4.64 (5)
Perceived Usefulness	5.68 (5.5)	6.41 (6.5)	5.50 (6)
Perceived Ease of Use	4.73 (4.5)	5.95 (6)	4.59 (4.5)
Behavioral Intention	5.82 (6)	6.32 (6.5)	5.64 (6)
Perceived External Control	5.36 (6)	6.00 (6)	5.55 (5)

Note. ST, Single Task and DT, dual task.

#### 3.2.3 *Ad hoc* wellbeing and working experience

No differences emerged considering all the dimensions (all *p*_s_ > 0.05). Mean and median (in parentheses) scores are reported in Table 4. Wellbeing and working experience questionnaire (scale median = 3; 5-point scale, not at all-extremely).

**TABLE 4:** Wellbeing and working experience questionnaire dimensions and scores.

WB Dimensions	Pre-test	Post ST	Post DT
	Mean (Median)	Mean (Median)	Mean (Median)
Motivation	3.14 (3.5)	3.82 (4)	3.50 (3.5)
Engagement	3.18 (3)	3.64 (4)	4.05 (4)
Satisfaction	2.91 (3)	3.91 (4)	3.03 (3)
Work/Task Experience	2.86 (3)	3.59 (3.5)	3.12 (3)

Note. ST, Single Task and DT, dual task.

#### 3.2.4 Social impact

**FIGURE 8:**
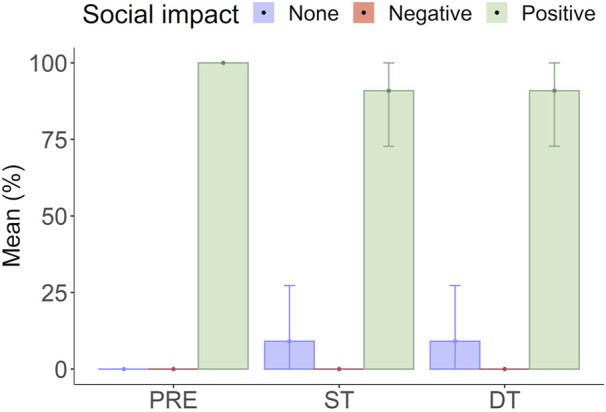
Mean responses (%) concerning the perceived social impact of the AAS as a function of the experimental phase. Note: PRE, Pre-test; ST, Single Task; DT, Dual task.

Overall, participants reported a positive perception regarding the potential effect of introducing an AAS equipped with a cobot in an Industrial context. Indeed, more than 90% of the participants at pre-test and both post-tests choose “it will positively affect the working activities, but it will not cause the dismissal of workers.” Only 10% of participants in both post-tests selected “it will not produce effects on working activities” (Figure 8).

### 3.3 Eye tracking

#### 3.3.1 Fixation duration

Considering fixations duration, three t-tests were performed. No difference emerged between single and dual task conditions (*p* > 0.05; respectively, single task: *M* = 142.15 ms; dual task *M* = 139.47 ms). Differently, both experimental conditions (resting vs. single task: *t* = 5.43, *df* = 10, *p* < 0.001, *d* = 2.47; resting vs. dual task: *t* = 5.96, *df* = 10, *p* < 0.001, *d* = 2.58) showed a significant reduction in the average duration of fixations compared to the resting condition (*M* = 325.50 ms).

#### 3.3.2 Fixation frequency

Pertaining to the frequency of fixations, a series of t-tests did not highlight any difference between single and dual tasks. The mean fixation frequency per minute was similar among the resting phase and the experimental conditions (resting: *M* = 140.49; single task: *M* = 146.00; dual task: *M* = 147.21).

#### 3.3.3 Blink duration

Regarding the blink duration, t-tests were carried out. No difference was shown between the experimental conditions (*p* > 0.05; respectively, single task: *M* = 379.78 ms; dual task: *M* = 424.69 ms). Nonetheless, in both experimental conditions (resting vs. single task: *t* = −3.06, *df* = 10, *p* < 0.05, *d* = 1.79; resting vs. dual task: *t* = −2.39, *df* = 10, *p* < 0.05, *d* = 0.97), the duration of blink was longer than in the resting condition (*M* = 239.65 ms).

#### 3.3.4 Blink frequency

**FIGURE 9:**
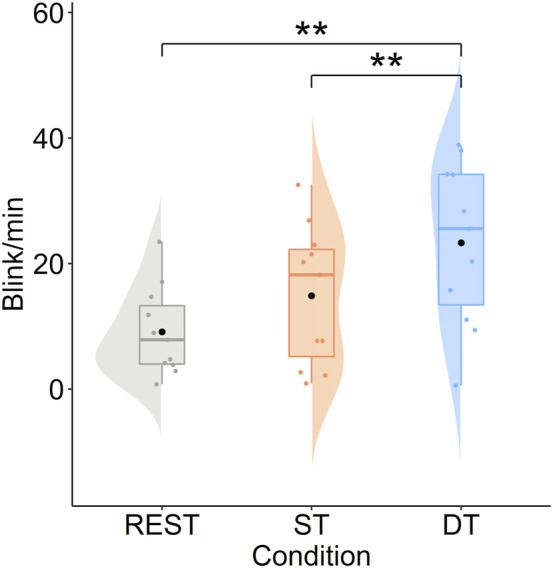
Mean blink frequency/min as a function of condition. Note: REST, Baseline resting condition; ST, Single Task; DT, Dual Task.

Concerning the blink frequency, a difference was underlined (*t* = −5.03, *df* = 10, *p* < 0.01, *d* = 0.70) between experimental conditions. Participants blinked more frequently in the dual task (*M* = 23.30 blink/min) compared to the single task (*M* = 14.87 blink/min; see Figure 9). Besides, only the dual task condition differed from the resting conditions (resting: *M* = 9.12 blink/min; resting vs. dual task: *t* = −4.84, *df* = 10, *p* < 0.01, *d* = 1.37).

### 3.4 Cardiac activity

No difference in heart rate was shown between single and dual task conditions (*p* > 0.05). The average heart rate was similar in the experimental sessions (single task: *M* = 101.25 bpm; dual task: *M* = 100.05 bpm), while, both conditions differed from the resting phase (resting: *M* = 78.05; resting vs. single task: *t* = −5.50, *df* = 10, *p* < 0.001, *d* = 1.78; resting vs. dual task: *t* = −3.01, *df* = 10, *p* < 0.05; *d* = 1.31).

### 3.5 Multiple linear regressions

**FIGURE 10:**
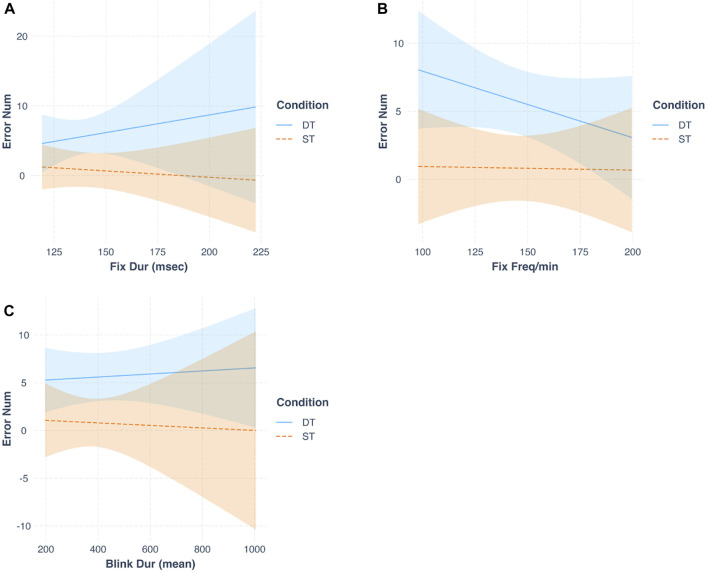
Multiple linear regressions showing the predicting power of Fixation duration **(A)**, Fixation Frequency **(B)**, Blink duration **(C)** over the Number of errors in the two experimental conditions. Note: DT, Dual task; ST, Single Task; Fix Dur, Fixation Duration; Fix Freq/min, Fixation Frequency; Blink Dur, Blink Duration.

A first multiple linear regression analysis was carried out to assess if the implicit measures (i.e., time on task, fixation duration, fixation frequency, blink duration, blink frequency, and heart rate) could predict task accuracy in the assembly task (explicit measure) including in the model also all the interactions between the implicit measures and the condition. This model (m1) was overall not significant [F (13, 8) = 2.41, *p* = 0.11, *R* ^2^ = 0.47], although some of the predictors and interactions were significant. For this reason, we refined the model (m2) by removing the variables (i.e., time on task, heart rate) and the corresponding interactions that were not contributing to the predictive power of the model (James et al., 2013). The second model was significant (F (9, 12) = 4.68, *p* < 0.01, *R* ^2^ = 0.61). Besides, the reduction in the residual standard error from m1 to m2 (respectively from 3.24 to 2.76) suggested that m2 is better fitting the data. We further analyzed the individual predictors. The fixation duration (B = 0.33, *t* = 3.78, *p* < 0.01), fixation frequency (B = −0.21, *t* = −4.40, *p* < 0.001), blink duration (B = 0.02, *t* = −3.98, *p* < 0.01), and blink frequency (B = 0.23, *t* = 2.84, *p* < 0.05) were able to predict the accuracy in the assembly task significantly. Besides, three significant interactions emerged (Figure 10): fixation duration X condition (B = −0.34, *t* = −3.75, *p* < 0.01), fixation frequency X condition (B = 0.21, *t* = 3.72, *p* < 0.01), and blink duration X condition (B = 0.02, *t* = 2.7, *p* < 0.05).

We carried out a second a multiple linear regression analysis to predict the task performance in terms of time on task on the basis of the implicit metrics including in the model all the interactions between the implicit measures and the condition. The first model was overall not significant [F (13, 8) = 1.13, *p* = 0.44, *R* ^2^ = 0.08], and all the predictors and interactions were not significant (all *p*_*s*_ > 0.30).

Besides, a series of multiple regressions was performed to analyze if the implicit measures could predict the scores assigned to the different NASA-TLX subscales. The outcomes of the first regression model, which considered mental demand as the dependent variable, did show a significant collective effect between the considered predictors [F (15, 6) = 7.64, *p* < 0.01, *R* ^2^ = 0.83]. Nonetheless, the predictors or their interactions with the condition did not predict the mental demand scores (all *p*_*s*_ > 0.05). Considering the other multiple linear regressions they did not show collective effects [i.e., physical demand: F (15, 6) = 0.97, *p* > 0.05, *R* ^2^ = −0.02; temporal demand: F (15, 6) = 1.87, *p* > 0.05, *R* ^2^ = 0.38; performance: F (15, 6) = 1.72, *p* > 0.05, *R* ^2^ = 0.34; effort: F (15, 6) = 2.23, *p* > 0.05, *R* ^2^ = 0.47; frustration: F (15, 6) = 3.41, *p* > 0.05, *R* ^2^ = 0.63].

## 4 Discussion

The present experiment aimed at a thorough analysis of a series of human factors in a cutting-edge manufacturing setting, which involved an advanced ergonomic workstation and a cobot. By following the Industry 5.0 conceptualization, we proposed a human-centered study. We specifically targeted senior workers, as this population is particularly inclined to a decrement in their working abilities and, therefore, would particularly benefit from the introduction of supportive and collaborative systems such as cobots in their daily work life (Bogataj et al., 2019). The main objective of this study was thus to provide a broad assessment of various human factors (e.g., senior workers’ mental workload and task accuracy) during the execution of an assembly task in collaboration with a cobot, installed on an assistive assembly workstation. More specifically, the following human factors were analyzed: task performance (i.e., number of errors and time on task), subjective perceptions (i.e., the perceived workload reported at the NASA-TLX, the cobot acceptance assessed via TAM, the level of wellbeing and work experience, the social impact of using cobots), eye tracking indices (i.e., blink and fixation frequency and duration), and the cardiac activity. A dual task paradigm was used to manipulate the task difficulty, and therefore, the participants’ mental loads.

As a first objective, we wanted to provide a broad assessment of human perceptions regarding the integration of a cobot within a work environment, including technology acceptance, wellbeing, and working experience as well as the broader social impact of this integrated technology in the industrial sector. Our senior participants’ scores were high (>4.3) above the scale median (4) both after the single task and after the dual task condition, demonstrating that they enjoyed working with the AAS, they found it useful and easy to use, they expressed the intention to use it if available, they perceived to have control over the system, and they possess the necessary skills to utilize it. Furthermore, at both post-tests, the wellbeing and work experience scores (>3) showed high reported motivation, engagement, satisfaction, and positive work/task experience. Finally, most participants reported that the AAS would have a positive effect on the working activities and would not cause the dismissal of workers if implemented in a real-world scenario. It is important to highlight that these findings were observed in older workers, who might have less experience and skills with advanced technologies compared to a younger population. This observation is in line with Rossato et al. (2021a), who found that older workers viewed the cobot as being more helpful than a group of younger adult workers did.

As a second objective, we aimed at evaluating if the human factors examined in this study (e.g., task performance, subjective perceptions, eye tracking measures, and cardiac activity) significantly changed during dual tasking with increased mental load. On this regard, the level of AAS acceptance, wellbeing, and work experience scores did not differ in the dual task compared to the single task. These findings thus suggest that the cobot was actually supportive and well-accepted even during dual tasking when handling a new technology while under mental strain could have introduced an additional challenge.

Concerning the performance measures, as predicted, both performance indices were modulated. Indeed, the increment in difficulty (dual task; i.e., assembly task + concurrent mathematical task) resulted in a higher number of errors and a longer time on task compared to the condition in which participants had to accomplish the assembly task only. These results align with previous literature using dual tasking to increase task difficulty (Galy and Mélan, 2015; Shaw et al., 2018; Vasquez et al., 2019).

Furthermore, as regards the perceived workload, participants showed a higher level of perceived mental demand and effort while accomplishing the dual task. This result confirms that our manipulation successfully also increased the perceived level of mental demand in the users, and it is in line with previous research (Rubio et al., 2004; Mansikka et al., 2019; Mingardi et al., 2020; Lowndes et al., 2020; Panchetti et al., 2023). Concerning the physical demand, the absence of single vs. dual task difference simply due to the nature of the secondary task being predominantly cognitive (i.e., mathematical), did not affect participants’ perception in terms of physical strain. Regarding the temporal demand, a difference was not shown insofar as senior workers were expected to execute the tasks in the various experimental condition with both speed and accuracy, albeit without adhering to a predefined time constraint. Participants reported a similar level of perceived performance, suggesting that they may not have been aware of the disparity in difficulty and, as a result, inadvertently committed more errors in the dual task condition. Finally, the dual task condition was not associated with a higher level of perceived frustration. This finding also substantiates the lack of awareness regarding their actual performance in the two conditions. In fact, being conscious of committing more errors in the dual task would have been expected to be related to a higher sense of frustration. In the context of advanced workstations and collaborative robotics, failing to recognize performance deterioration can result in increased operational risks, higher error rates, compromised quality control, and adverse effects on workers’ health and wellbeing. Therefore, it is crucial to explore and study the implementation of monitoring technologies that can quickly identify performance decline, especially in individuals who may be more prone to it due to factors like age. This area deserves further investigation in future research.

Regarding the implicit measures, instead, we did highlight a difference in one of the eye behavior metrics. Indeed, blink frequency associated with the dual task condition was higher than in the single task condition. It thus seems that, based on previous literature (Faure et al., 2016; Tao et al., 2019; Mingardi et al., 2020), participants experienced a higher level of mental load in the dual task compared to the single task. Nonetheless, the fixation frequency and duration, as well as the blink duration and also cardiac activity, did not demonstrate to change significantly under dual tasking. On this matter, it is possible that these indices were not sensitive to the mental load fluctuations during our assembly task, while they demonstrated to be sensitive to mental load fluctuations in different work tasks (e.g., a manual screwing task, Mingardi et al., 2020). This generates new questions about whether the psychophysiological indices’ sensitivity to mental load in such ecological work contexts is task-dependent, a question that is worth investigating in future research.

Finally, we investigated the predictive capacity of the gathered measurements on task-related errors and perceived mental demand. Our results from the first linear regression analyses demonstrated how all the measured eye behavioral indices (i.e., fixation duration and frequency, and blink duration and frequency) successfully predicted the number of errors committed at the assembly task. Interestingly, these indices had a stronger predictive power on the committed errors in the dual task condition compared to the single task one, suggesting that the higher the mental demand, the more these indices differ with varying error rates at the task. This could be related to the fact that eye blinks and fixations are known to respond to different levels of task complexity and mental demand (Matthews et al., 2015; Mingardi et al., 2020; Wu et al., 2020; Nenna et al., 2023). Therefore, even though we only found a significant effect of dual tasking over blink frequency, the eye indices might show significant modulations under higher mental strain particularly. More specifically, we found that an increase in the number of errors committed at the assembly task is related to an increase in the fixation duration and the blink frequency, and with a decrease in the fixation frequency. Future works might extend research on the predictive power of eye indices over work task accuracy, similarly to what was done with workload estimation (e.g., Novak et al., 2015), and consider the possibility of implementing eye measures to actively predict the increase rate of task errors online, during the interaction with cobots.

Otherwise, the second series of linear regression analyses showed that task accuracy and the implicit measures were not capable of predicting the scores of the NASA-TLX sub-scales. A suitable explanation is that participants were not aware of their actual performance. Indeed, differences in both task accuracy and time on task (i.e., higher error rates and longer time in the dual task condition) were not related to a discrepancy in the NASA-TLX sub-scale of perceived performance. This outcome is very relevant regarding work safety insofar as not being aware of a decline in performance due to a more demanding working activity could potentially be related to a diminution of overall attention and the adoption of unsafe behaviors.

Some limitations of the study could be underlined. Firstly, we considered a small sample size (*N* = 11), so we must exercise caution when generalizing the findings of the current study. Secondly, more ecological tasks must be considered, especially in terms of duration that could be similar to a phase of a real working shift to also have reliable data, for instance, to assess work-related stress exploiting heart-rate variability (HRV; Gervasi et al., 2020).

Overall, this paper contributes to the literature by proposing a human-centric perspective and a thorough analysis of various human factors to shed light on the feasibility of integrating advanced ergonomic workstations and cobots within industrial manufacturing contexts. While the benefits of these technologies for industrial production are well-known, our study uniquely examines their impact on human factors by adopting a multi-method approach that includes various data sources (performance, self-report, eye-tracking and heart rate data). In a future perspective, the relationships between implicit measures acquired while participants were performing the tasks (e.g., eye-tracking indices) and the working performance could be exploited to inform advanced workstations equipped with wearable sensors (e.g., eye trackers, chest bands) that could adapt their functioning based on the detection of variations in the level of mental load (i.e., overload), with the intention of assisting the workers when they are dealing with more mentally demanding working activities. For instance, future directions might involve adapting these systems for the effective detection and mitigation of worker overload states in diverse industrial environments. This may encompass the development of tailored interventions and the integration of adaptive technologies to enhance worker wellbeing and productivity while maintaining safety standards. However, to implement such a flexible system, it is first imperative to understand human needs. In this respect, we here assessed technology acceptance and perceived wellbeing among senior workers, shedding light on their experiences with these integrated technologies in industrial settings. This holistic approach advances our understanding of the complex interplay between humans and technology, paving the way for safer, more inclusive, and efficient working environments in the evolving manufacturing landscape.

## Data Availability

The raw data supporting the conclusion of this article will be made available by the authors upon specific request.
